# Aberrant SSEA-4 upregulation mediates myofibroblast activity to promote pre-cancerous oral submucous fibrosis

**DOI:** 10.1038/srep37004

**Published:** 2016-11-15

**Authors:** Cheng-Chia Yu, Chuan-Hang Yu, Yu-Chao Chang

**Affiliations:** 1 Institute of Oral Sciences, Chung Shan Medical University, Taichung, Taiwan; 2 School of Dentistry, Chung Shan Medical University, Taichung, Taiwan; 3 Department of Dentistry, Chung Shan Medical University Hospital, Taichung, Taiwan

## Abstract

Oral submucous fibrosis (OSF), regarded as a precancerous condition, is characterized by juxta-epithelial inflammatory reaction followed by fibro-elastic change in the lamina properia and epithelial atrophy. The pathologic mechanisms of OSF still need to be further clarified. In the study, we investigated the functional expression of SSEA-4, which is a well-known stemness marker, in myofibroblast activity and the clinical significance in OSF tissues. The expression of SSEA-4 in OSF was evaluated by immunohistochemical staining. Functional analysis of SSEA-4 on myofibroblast activity of OSF was achieved by lentiviral silencing ST3GAL2. Immunohisitochemistry demonstrated that SSEA-4 expression was significantly higher expression in areca quid chewing-associated OSF tissues than those of normal oral mucosa tissues. From flow cytometry analysis, arecoline dose-dependently activated SSEA-4 expression in primary human normal buccal mucosal fibroblasts (BMFs). Sorted SSEA-4-positive cells from fibrotic BMFs (fBMFs) have higher colony-forming unit, collagen gel contraction, and α-smooth muscle actin (α-SMA) expression than SSEA-4-negative subset. Knockdown of ST3GAL2 in fBMFs suppressed SSEA-4 expression, collagen contraction, migration, invasiveness, and wound healing capability. Consistently, silencing ST3GAL2 was found to repress arecoline-induced myofibroblast activity in BMFs. The study highlights SSEA-4 as a critical marker for therapeutic intervention to mediate myofibroblast transdifferentiation in areca quid chewing-associated OSF.

Oral submucous fibrosis (OSF), a chronic progressive scarring disease which characterized by the submucosal accumulation of dense fibrous connective tissue with inflammatory cell infiltration, has been considered as pre-cancerous condition^1^. Based on the epidemiological evidence, areca quid chewing is the most important etiological factor for OSF. However, the pathogenic mechanisms of areca nut constituents in areca quid chewing-associated OSF have not been well addressed. Previous studies demonstrated that myofibroblast could play an important role in the pathogenesis of fibrosis^2^. Up-regulation of myofibroblast activity has been found in several organ fibrosis, such as liver^3^, heart^4^, lung^5^, and OSF^6^,^7^. The expression of α-smooth muscle actin (α-SMA) is positively correlated with severity in OSF tissues^6^. Arecoline, the major areca nut alkaloid, could activate the transdifferentiation of oral keratinocytes into myofibroblasts^8^. Previously, we have also identified that the treatment of arecoline could induce myofibroblasts activities in human normal buccal mucosal fibroblasts (BMFs)^9^,^10^,^11^, supporting the crucial roles of myofibroblasts transdifferentiation during the pathogenesis of OSF.

Stage-specific embryonic antigen-4 (SSEA-4), a sialyl-glycolipid, has been shown to play an important role in both stem cell and cancer biology^12^,^13^. SSEA-4 is well-known cell surface marker for embryonic stem cells and pluripotent stem cells^14^. SSEA-4 is also frequently overexpressed in cancer cells and cancer stem cells^15^. SSEA-4 is bio-synthesized from SSEA-3 by β-galactoside α-2,3-sialyltransferase 2 (ST3GAL2). Knockdown of ST3GAL2 impairs cell adhesion and extracellular cell matrix-related molecules including collagen I, collagen IV, chondroitin sulfate, and laminin in DU145 cells^16^. ST3GAL2 overexpression is a predictor for poor prognosis of breast and ovarian cancer patients^12^. Immunohistochemistry analysis showed that ST3GAL2 expression is highly expressed in high-grade glioblastoma specimens, compared with the normal brain tissue^17^. CD44^+^ SSEA-4^+^ oral cancer cells display the characteristics of self-renewal and high tumorigenicity both *in vitro* and *in vivo*^15^. Interestingly, sorted SSEA-4^+^ fibroblasts subpopulation from idiopathic pulmonary fibrosis could display mesenchymal stem cells and fibrogenic characteristics^18^. However, the functional role of SSEA-4 in OSF still remains unknown.

In this study, we first demonstrate that SSEA-4 expression is significantly increased in areca quid chewing-associated OSF tissues. Arecoline could induce SSEA-4 expression in BMFs. Knockdown of SSEA-4 synthesis could abolish myofibroblast activity in fibrotic BMFs (fBMFs). Therefore, targeting SSEA-4 may be a potential therapeutic approach to suppress myofibroblast transdifferentiation for the pathogenesis of areca quid chewing-associated OSF.

## Results

### Elevation of SSEA-4 expression in OSF specimens

**Figure 1:**
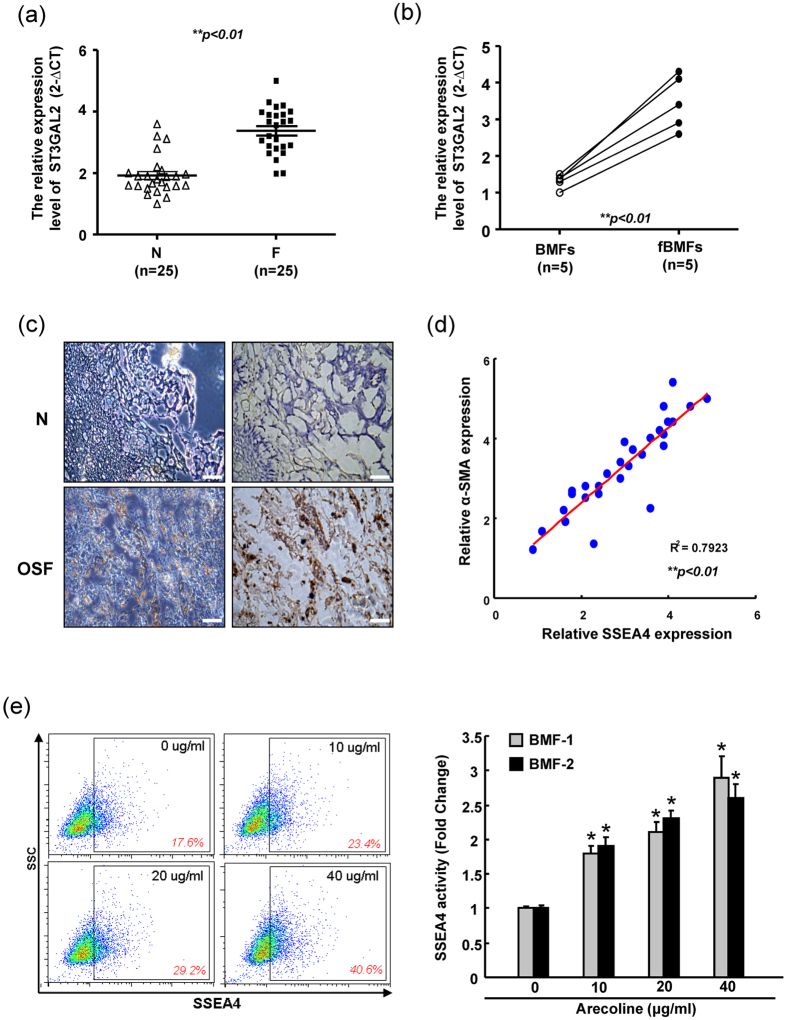
Up-regulation SSEA-4 expression in OSF. (**a**) Normal buccal mucosa (N) tissues and fibrotic buccal mucosa (F) lesions were subjected to real-time RT-PCR analysis for the determination relative expression levels of ST3GAL2. (**b**) Real-time RT-PCR analysis demonstrated that ST3GAL2 expression in independent pairs (n = 5) of normal human buccal mucosal fibroblasts (BMFs) and human fibrotic buccal mucosal fibroblasts (fBMFs). (**c**) Positive correlations between SSEA-4 and α-SMA in OSF tissues were analyzed. (**d**) Representative images of normal buccal mucosa (N) tissues and OSF specimen lesions were subjected to histological analysis for the expression levels of SSEA-4. (**e**) BMFs were serum-starved (0.5% FBS) for 48 h and treated with indicated concentrations of arecoline for further 24 h in serum-free medium. The SSEA-4 activity in arecoline-treated BMFs was assessed by flow cytometry and presented as relative fold-changes. *Represents significant difference from control values with *p* < 0.05.

To elucidate the clinical significance of SSEA-4 in OSF specimens, we compared the relative expression levels of ST3GAL2 (the rate-limiting enzyme of SSEA-4 synthesis) and SSEA-4 expression between normal buccal mucosa (N) and fibrotic buccal mucosa (F) from OSF ttissues. Initially, real-time RT-PCR analysis demonstrates that mRNA level of ST3GAL2 was higher in F samples but lower in N subjects (Fig. 1a). An increase of ST3GAL2 in primary cultivated fibroblasts from OSF tissues in comparison with pair BMFs subjects (Fig. 1b). Clinical results revealed that ST3GAL2 was positively correlated with α-SMA in the OSF tissues (Fig. 1c). Histopathological evaluation showed that strong SSEA-4 staining in human OSF tissues compared with those in BMF tissues (Fig. 1d). Through examining the 35 OSF specimens, 82.8% and 80% of cases displayed strong expression of SSEA4 and α-SMA as compared with normal mucosal tissues, respectively (P < 0.05, Table 1). The pathogenesis of OSF is highly associated with areca quid chewing. To further examine the effect of arecoline, the major areca nut alkaloid, on SSEA-4 expression in BMFs, BMFs treated with arecoline and the levels of SSEA-4 were measured by flow cytometry analysis. Arecoline treatment dose-dependently elevated SSEA-4 expression in BMFs (Fig. 1e).

### SSEA-4^+^ fBMFs displayed stemness and myofibroblast properties

**Figure 2:**
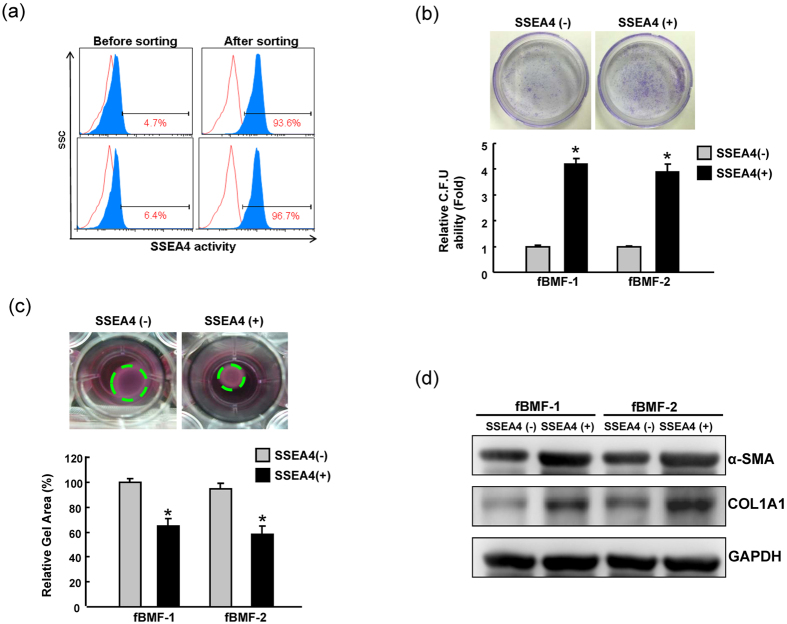
Increased of stemness and myofibroblast activity in SSEA-4^+^ fBMFs. (**a**) The expression of SSEA-4 in sorted fBMFs was analyzed by flow cytometry. To elucidate the capabilities of colony formation (**b**) and collagen contraction (**c**) of SSEA-4^+^ and SSEA-4^−^ fBMFs, single-cell suspensions of fBMFs were plated and analyzed as described in the experimental section. (**d**) Protein levels of α-SMA and COLA1 in SSEA-4^+^ and SSEA-4^−^ fBMFs were examined by western blotting analysis. The amount of GAPDH protein of different crude cell extracts was referred to as a loading control. The original blots are shown in Supplementary Fig. 1. Error bars correspond to SD. Data shown here are the mean ± SD of three independent experiments. (*p < 0.05).

The SSEA-4 surface stemness marker has been used to identify dental pulp stem cells and cancer stem cells^14^. With flow-activated cell sorting (FACS) cell sorter, SSEA-4^+^ and SSEA-4^−^ cells from human primary fBMFs were isolated and the sorted purity in fBMFs was further analyzed (Fig. 2a). The colony-forming efficiency of SSEA-4^+^ cells was significantly higher than those of the SSEA-4^−^ cells (Fig. 2b). As shown in Fig. 2c, collagen contractility was readily detectable in SSEA-4^+^ cells but was low or undetectable in SSEA-4^−^ cells. By western blotting analysis, we observed the increased expression of α-SMA and COL1A1 in SSEA-4^+^ cells compared that in SSEA-4^−^ cells (Fig. 2d, Supplementary Fig. 1). Taken together, we hypothesized that the upregulation of SSEA-4 might be crucial for modulating myofibroblast properties in fBMFs.

### ST3GAL2 down-regulation repressed collagen contractility in fBMFs

**Figure 3:**
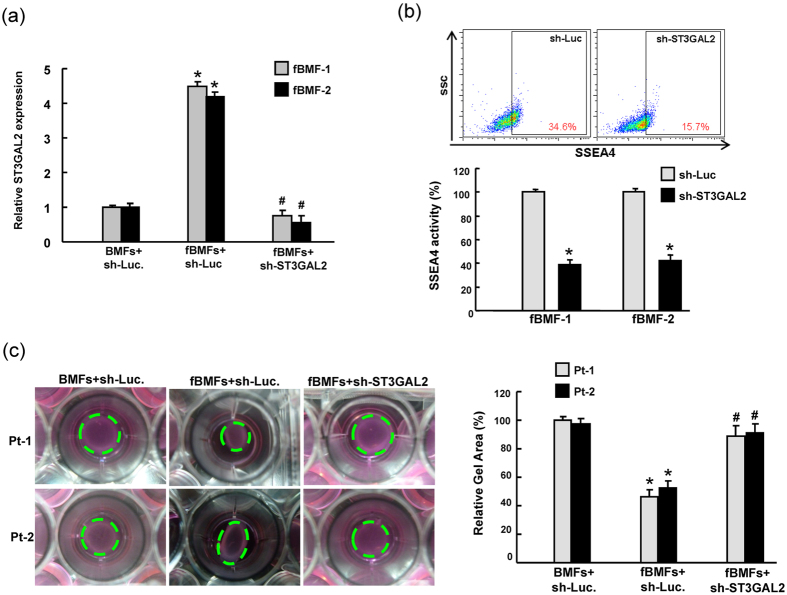
The effect of ST3GAL2 on SSEA-4 expression and collagen contractility in fBMFs. The silencing effect of SSEA-4 by ST3GAL2 knockdown in fBMFs was validated by real-time RT-PCR^−^ (**a**) and flow cytometry- (**b**). (**c**) fBMF were transduced with ST3GAL2 shRNA lentivirus and embedded into collagen gels. After 48 h, contraction of the gels was photographed and measured using ImageJ software (NIH) to calculate their areas (right panel).

To further investigate whether SSEA-4 could mediate myofibroblast properties in fBMFs, the approach of loss-of-function of ST3GAL2 was first conducted through lentiviral-mediated knockdown. The knockdown efficiency of ST3GAL2 in fBMFs was validated by real-time RT-PCR analysis (Fig. 3a). Flow cytometry analysis confirmed that silencing ST3GAL2 inhibited SSEA-4 activity in fBMFs (Fig. 3b). Furthermore, in vitro functional assays indicated that the down-regulation of ST3GAL2 significantly reduced collagen contraction capacities in fBMFs (Fig. 3c).

### Silencing ST3GAL2 inhibited myofibroblast properties and marker expression in fBMFs

**Figure 4:**
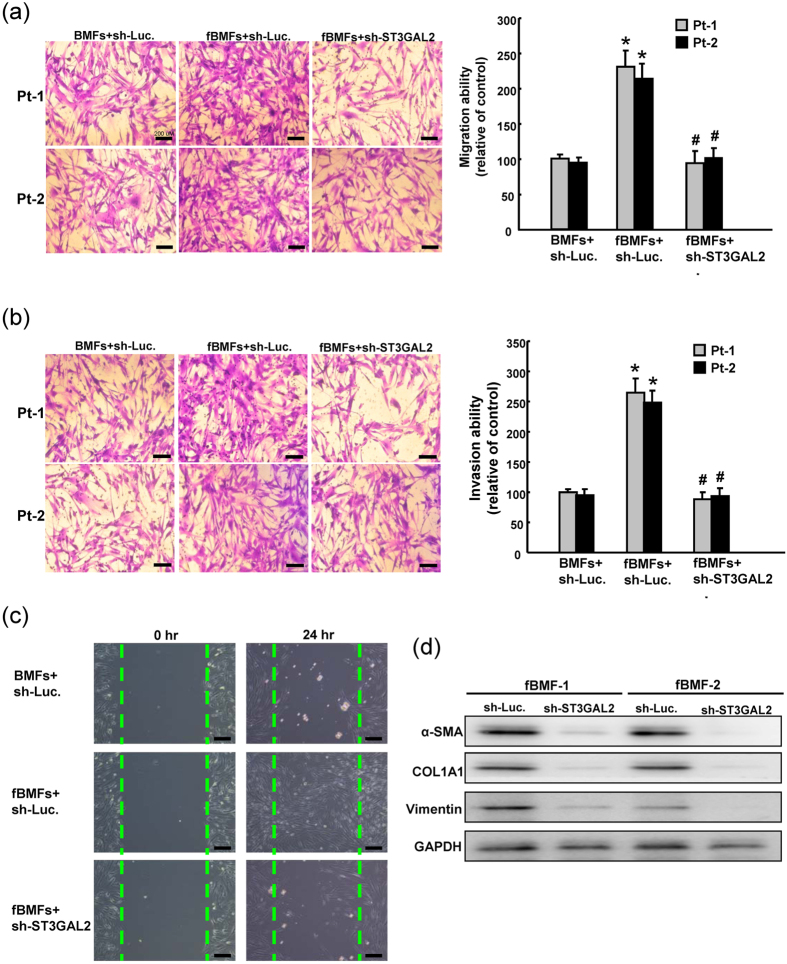
The effect of ST3GAL2 on myofibroblast activity and marker expression in fBMFs. The sh-Luc.-expressing and sh-ST3GAL2-expressing fBMFs were subjected to *in vitro* migration and invasion assay, and the number of migration (**a**) and invasive (**b**) cells was calculated and is presented as the fold-change relative to sh-Luc.-expressing cells. (**c**) Single cell suspension of fBMFs infected with ST3GAL2-specific shRNA or control sh-Luc lentivirus was analyzed by wound healing assay. (**d**) The protein expression levels of α-SMA and COLA1 in fBMFs infected with ST3GAL2-specific shRNA or control sh-Luc lentivirus were analyzed by western blot. The original blots are shown in Supplementary Fig. 2. *P < 0.05 Sh-Luc.+fBMFs group versus Sh-Luc.+BMFs group; ^#^P < 0.05 Sh- ST3GAL2 versus Sh-Luc. group.

To further determine the biological functions of SSEA-4 in myofibroblast activity, two fibroblast strains of fBMFs was knockdown by silencing ST3GAL2 expression and cell migration/invasion was monitored by using tanswell system. The migration (Fig. 4a) and invasion (Fig. 4b) capability of fBMFs was significantly higher than those of BMFs. Silencing ST3GAL2 in fBMFs was shown to reverse migration (Fig. 4a) and invasion (Fig. 4b) abilities in fBMFs. To further investigate associations between myofibroblast activity and SSEA-4, we analyzed the wound healing ability in control and ST3GAL2-knockdown fBMFs. The wound healing ability was elevated as compared with the control BMFs cells. Upon ST3GAL2 knockdown, the wound healing ability was decreased in fBMFs (Fig. 4c). Expression patterns of myofibroblast-associated makers including SMA, vimentin, and COLA1 were decreased in fBMFs with ST3GAL2 knockdown by western blotting (Fig. 4d, Supplementary Fig. 2).

### Silencing ST3GAL2 repressed arecoline-induced myofibroblast activities in BMFs

**Figure 5:**
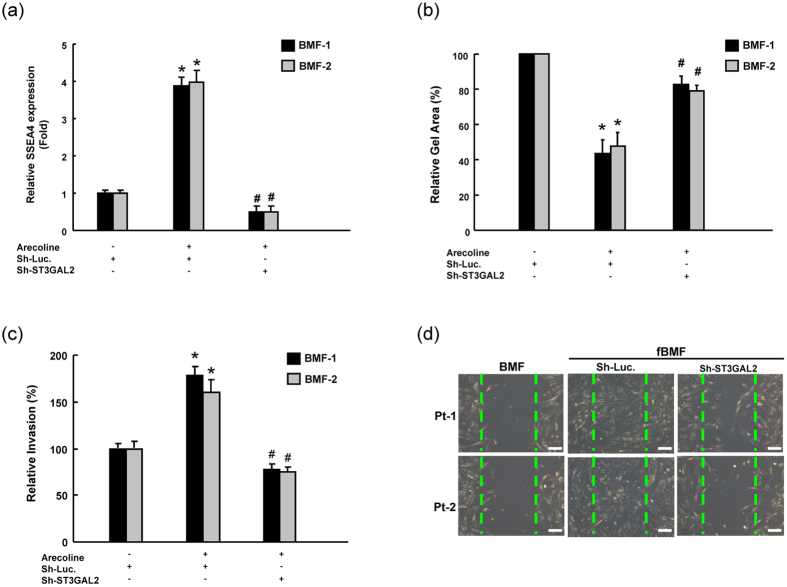
Depletion of ST3GAL2 repressed arecoline-induced myofibroblastic differentiation activity in BMFs. (**a**) The silencing effect of ST3GAL2 shRNA in arecloine-treated BMFs was validated by flow cytometry. Single cell suspension of arecoline-treated BMFs infected with ST3GAL2-specific shRNA or control sh-Luc lentivirus was analyzed for collagen gel contractility (**b**), migration ability (**c**), and wound healing ability (**d**). *P < 0.05 Sh-Luc.+arecoline group versus Sh-Luc. group; ^#^P < 0.05 Sh- ST3GAL2+arecoline or Sh-2+arecoline versus Sh-Luc.+arecoline group.

Our previous studies have demonstrated that arecoline could induce myofibroblast activities in BMFs^11^,^19^. To further investigate whether SSEA-4 plays a role in maintaining arecoline-induced myofibroblastic differentiation activity, loss-of-function of ST3GAL2 was conducted in arecoline-stimulated BMFs. Flow cytometry analyses confirmed that lentivirus expressing both sh-*ST3GAL2* markedly reduced the expression level of arecoline-induced SSEA-4 expression in BMFs (Fig. 5a). Consistently, ST3GAL2 knockdown abrogated arecoline-induced collagen gel contraction (Fig. 5b), invasion (Fig. 5c), and wound healing (Fig. 5d) abilities in BMFs.

## Discussion

Mounting evidences have demonstrated that the dys-regulation of stemness marker expression contributes to the pathogenesis of diseases, especially its roles in tumorigenesis^20^,^21^. Some aberrant stemness marker expressions such as Bmi1, CD133, or Sox2 have been found to be involved in the progression of fibrosis. Hypoxia activated HIF-1α/Twist-Bmi1 axis promote epithelial-mesenchymal transition (EMT) and renal fibrogenesis^22^. CD133-positive cells could drive chronic and acute phases of primary myelofibrosis in mice^23^. Sox2-positive skin progenitor cells were found to contribute to bleomycin-induced skin fibrosis^24^. Increased Oct-4 and SSEA-4 expressions are demonstrated in keloid tissues compared to normal compartments^25^. These reports prompt us to examine whether SSEA-4, a well-known stemness marker, might play an important role in OSF. In this study, we first found that the increased SSEA-4 expression is demonstrated in OSF tissues and arecoline-stimulated BMFs (Fig. 1). Functional lentiviral-mediated knockdown ST3GAL2 could inhibit myofibroblastic differentiation activities and markers expression in fBMFs (Fig. 4) and arecoline-stimulated BMFs (Fig. 5). Our data suggest that the up-regulation of SSEA-4 expression may involve in the pathogenesis of areca quid chewing-associated OSF.

The pathogenesis of OSF is regulated by a mechanism known as EMT^26^,^27^. Stromal fibroblasts or endothelial cells or from terminally epithelial differentiated cells that undergo an EMT process to transdifferentiate myofibroblasts. Up-regulation of EMT-related molecules expression, such as plasminogen activator inhibitor-1 (PAI-1)^28^, insulin-like growth factor-1 (IGF-1)^29^, NF-κB^30^, vimentin^31^, S100A4^19^, or ZEB1^11^, is involved in the pathogenesis of OSF. Recently, our studies have demonstrated that ZEB1, a well-known factor in activation of EMT program, binds to the α-SMA promoter and transdifferentiate fibroblasts into myofibroblasts^11^. Numerous key profibrotic cytokines components of EMT have been identified, such as transforming growth factor-β (TGF-β), platelet-derived growth factor, and IGF-1^32^. TGF-β, a multifunction cytokine, plays crucial roles in EMT program and fibrosis^33^,^34^,^35^,^36^. Arecoline could promote the transdifferentiation of human BMFs into myofibroblasts through activating integrin αvβ6/TGF-β1 signaling^8^. Areca nut extracts were found to induce TGF-β signaling in primary human gingival fibroblast^37^. Recently, flow cytometry analysis has demonstrated that the treatment of TGF-β1 could activate SSEA-4 expression leading to promote EMT program in breast cancer cells^12^. It is worthy to investigate that whether SSEA-4 up-regulation is mediated by TGF-β signaling and further manipulate the TGF-β signaling to block SSEA-4 expression and myofibroblastic differentiation.

Conclusively, this study presents the functional role of SSEA-4, a stemness marker, emphasizing its roles in myofibroblast properties during the pathogenesis of OSF. Clinical studies presented OSF tissue with increased expression of SSEA-4 as compared with normal oral mucosal tissues. Therefore, targeting SSEA-4 by ST3GAL2 knockdown ablates myofibroblast transdifferentiation activities and markers expression. The studies support the important role of SSEA-4 in the pathogenesis of areca quid chewing-associated OSF. SSEA-4 could be a therapeutic target for OSF.

## Materials and Methods

### Chemicals and reagents

Arecoline and collagen solution from bovine skin were purchased from Sigma-Aldrich (St. Louis, MO, USA).

### OSF tissues acquirement and immunohistochemistry

Before commencing the study, approval was obtained from the Institutional Review Board of Chung Shan Medical University Hospital, and informed written consent was obtained from each individual (CSMUH No: CS2-16009). All the methods applied in this study were carried out in accordance with the approved guidelines. For immunohistochemistry, formalin-fixed, paraffin-embedded specimens of 15 normal buccal mucosa from non-areca quid chewers, and 35 OSF specimens from areca quid chewers were collected in Department of Dentistry, Chung Shan Medical University Hospital. Tissue samples were spotted on glass slides for immunohistochemical staining. After deparaffinization and rehydration, tissue sections were processed with antigen retrieval by 1X Trilogy (Biogenics, Napa, CA, USA) diluted in H_2_O with heating. The slides were immersed in 3% H_2_O_2_ for 10 minutes and washed with PBS three times. Tissue sections were blocked with serum (Vestastain Elite ABC kit, Vector Laboratories, Burlingame, CA, USA) for 30 minutes, then incubated with the primary SSEA4 antibody. Diaminobenzidine (DAKO, Carpinteria, CA, USA) was then used as the substrate for localizing the antibody binding. Negative controls included serial sections from which either the primary or secondary antibodies were excluded. The preparations were counterstained with hematoxylin, mounted with Permount (Merck, Darmstadt, Germany), and examined by light microscopy^11^.

### Primary BMFs and fBMFs culture

Fibroblasts derived from normal buccal mucosa (BMFs) and fibrotic buccal mucosa (fBMFs) were cultured according to previous criteria and methods^19^. Cell cultures between the third and eighth passages were used in this study^19^.

### Quantitative real-time PCR (qRT-PCR)

Total RNA is prepared from cells using Trizol reagent according to the manufacturer’s protocol (Invitrogen Life Technologies, Carlsbad, CA, USA). qRT–PCRs of mRNAs are reverse-transcribed using the Superscript III first-strand synthesis system for RT–PCR (Invitrogen Life Technologies, Carlsbad, CA, USA). qRT-PCR reactions on resulting cDNAs were performed on an ABI StepOne™ Real-Time PCR Systems (Applied Biosystems)^20^. The primer sequences listed below:

*ST3GAL2*: 5′-TGGACGGGCACAACTTCATC-3′ and 5′-GGGCAGGTTCTTGGCACTCT-3′; *Gapdh*: 5′-CATCATCCCTGCCTCTACTG-3′ and 5′-GCCTGCTTCACCACCTTC-3′.

### Flow Cytometry analysis

Cells are stained with anti-SSEA-4 primary antibody and conjugated to secondary phycoerythrin (Miltenyi Biotech., Auburn, CA, USA) antibody, with labeling according to the manufacturer’s instructions. Red (>650 nm) fluorescence emission from 10,000 cells illuminated with blue (488 nm) excitation light is measured with a FACSCalibur (Becton Dickinson, Franklin Lakes, NJ, USA) using CellQuest software (Becton Dickinson, San Jose, CA, USA)^20^.

### Western blot analysis

Western blot analysis was performed by previously described protocols^20^. The primary antibodies will be those against α-SMA (1A4), vimentin (9E7E7), and rabbit ployclonal anti-human COL1A1 antibody were purchased from Santa Cruz Biotechnology, Inc. (Santa Cruz, CA, USA)^20^.

### Migration and invasion assays

These assay approaches have been well established in our laboratory and described as previously^38^.

### Lentivirus-based sh-ST3GAL2 knockdown

ST3GAL2 small hairpin RNA (ST3GAL2 sh-RNA)-expressing lentivirus or control construct was purchased from Sigma-Aldrich. Lentivirus production was performed by transfection of plasmid DNA mixture with lentivector plus helper plasmids (VSVG and Gag-Pol) into 293T cells using Lipofectamine 2000 (Invitrogen, Carlsbad, CA, USA). Supernatants were collected 48 h after transfection and then were filtered. Subconfluent cells were infected with lentivirus in the presence of 8 μg/ml polybrene (Sigma-Aldrich, St. Louis, MO, USA).

### Collagen contraction assay

Cells were suspended in 0.5 ml of 2 mg/ml collagen solution (Sigma-Aldrich, St. Louis, MO, USA) and added into one well of 24-well-plate. Plate was incubated at 37 °C for 2 hours which caused polymerization of collagen cell gels. After detaching gels from wells, the gels were further incubated in 0.5 ml medium for 48 h. Contraction of the gels was photographed and measured using ImageJ software (NIH, Bethesda, MD, USA) to calculate their areas^19^.

### Wound healing assay

Cells were seeded into a 12-well culture dish, and then wounds were introduced to the confluent monolayer of cells with a sterile 200 μL plastic pipette tip to create a denuded area. Cell movement into the wound area was photographed at 0 and 24 h under a microscope^39^.

### Statistical analysis

Data are presented as mean ± SD. A Student’s *t* test or analysis of variance (ANOVA) test was used to compare the continuous variables between groups, as appropriate. The chi-square test or Fisher’s exact test was used to compare the categorical variables. *P* < 0.05 was considered statistically significant.

## Additional Information

**How to cite this article**: Yu, C.-C. *et al*. Aberrant SSEA-4 upregulation mediates myofibroblast activity to promote pre-cancerous oral submucous fibrosis. *Sci. Rep.* **6**, 37004; doi: 10.1038/srep37004 (2016).

**Publisher’s note**: Springer Nature remains neutral with regard to jurisdictional claims in published maps and institutional affiliations.

## Supplementary Material

Supplementary Information

## Figures and Tables

**Table 1:** SSEA-4 and α-SMA expression in OSF tissues.

	Case Number	Weak	Strong	*P* value
SSEA-4				
Normal	15	9	6	
OSF	35	6	29	0.004
α-SMA				
Normal	15	11	4	
OSF	35	7	28	0.0302

SSEA-4 or α-SMA expression in human normal buccal mucosa or OSF was determined by immunohistochemistry as described in Materials and methods section. *P* value was calculated by Fisher exact test.

## References

[b1] Tilakaratne W. M., K. M. F., Saku Takashi, Peters T. J. & W. S. Oral submucous fibrosis: Review on aetiology and pathogenesis. Oral oncology 42, 561–568 (2006). 16311067 10.1016/j.oraloncology.2005.08.005

[b2] Wynn T. A. & Ramalingam T. R. Mechanisms of fibrosis: therapeutic translation for fibrotic disease. Nature medicine 18, 1028–1040, doi: 10.1038/nm.2807 (2012). PMC3405917 22772564

[b3] Kisseleva T. . Myofibroblasts revert to an inactive phenotype during regression of liver fibrosis. Proceedings of the National Academy of Sciences of the United States of America 109, 9448–9453, doi: 10.1073/pnas.1201840109 (2012). 22566629 PMC3386114

[b4] van den Borne S. W. . Myocardial remodeling after infarction: the role of myofibroblasts. Nature reviews. Cardiology 7, 30–37, doi: 10.1038/nrcardio.2009.199 (2010). 19949426

[b5] Hinz B. . The myofibroblast: one function, multiple origins. The American journal of pathology 170, 1807–1816, doi: 10.2353/ajpath.2007.070112 (2007). 17525249 PMC1899462

[b6] Angadi P. V., Kale A. D. & Hallikerimath S. Evaluation of myofibroblasts in oral submucous fibrosis: correlation with disease severity. Journal of oral pathology & medicine: official publication of the International Association of Oral Pathologists and the American Academy of Oral Pathology 40, 208–213, doi: 10.1111/j.1600-0714.2010.00995.x (2011). 21198872

[b7] Shi M. . Glucocorticoid regulation of a novel HPV-E6-p53-miR-145 pathway modulates invasion and therapy resistance of cervical cancer cells. The Journal of pathology 228, 148–157, doi: 10.1002/path.3997 (2012). 22287315

[b8] Moutasim K. A. . Betel-derived alkaloid up-regulates keratinocyte alphavbeta6 integrin expression and promotes oral submucous fibrosis. The Journal of pathology 223, 366–377, doi: 10.1002/path.2786 (2011). 21171082

[b9] Chang Y. C. . Resveratrol suppresses myofibroblast activity of human buccal mucosal fibroblasts through the epigenetic inhibition of ZEB1 expression. Oncotarget 7, 12137–12149, doi: 10.18632/oncotarget.7763 (2016). 26934322 PMC4914274

[b10] Lee Y. H. . Elevation of Twist expression by arecoline contributes to the pathogenesis of oral submucous fibrosis. Journal of the Formosan Medical Association = Taiwan yi zhi 115, 311–317, doi: 10.1016/j.jfma.2015.05.009 (2016). 26088962

[b11] Chang Y. C. . Arecoline-induced myofibroblast transdifferentiation from human buccal mucosal fibroblasts is mediated by ZEB1. Journal of cellular and molecular medicine 18, 698–708, doi: 10.1111/jcmm.12219 (2014). 24400868 PMC4000120

[b12] Aloia A. . The sialyl-glycolipid stage-specific embryonic antigen 4 marks a subpopulation of chemotherapy-resistant breast cancer cells with mesenchymal features. Breast cancer research: BCR 17, 146, doi: 10.1186/s13058-015-0652-6 (2015). 26607327 PMC4660783

[b13] Virant-Klun I., Kenda-Suster N. & Smrkolj S. Small putative NANOG, SOX2, and SSEA-4-positive stem cells resembling very small embryonic-like stem cells in sections of ovarian tissue in patients with ovarian cancer. Journal of ovarian research 9, 12, doi: 10.1186/s13048-016-0221-3 (2016). 26940129 PMC4778328

[b14] Gang E. J., Bosnakovski D., Figueiredo C. A., Visser J. W. & Perlingeiro R. C. SSEA-4 identifies mesenchymal stem cells from bone marrow. Blood 109, 1743–1751, doi: 10.1182/blood-2005-11-010504 (2007). 17062733

[b15] Noto Z. . CD44 and SSEA-4 positive cells in an oral cancer cell line HSC-4 possess cancer stem-like cell characteristics. Oral oncology 49, 787–795, doi: 10.1016/j.oraloncology.2013.04.012 (2013). 23768762

[b16] Sivasubramaniyan K. . Expression of stage-specific embryonic antigen-4 (SSEA-4) defines spontaneous loss of epithelial phenotype in human solid tumor cells. Glycobiology 25, 902–917, doi: 10.1093/glycob/cwv032 (2015). 25978997 PMC4565992

[b17] Lou Y. W. . Stage-specific embryonic antigen-4 as a potential therapeutic target in glioblastoma multiforme and other cancers. Proceedings of the National Academy of Sciences of the United States of America 111, 2482–2487, doi: 10.1073/pnas.1400283111 (2014). 24550271 PMC3932869

[b18] Xia H. . Identification of a cell-of-origin for fibroblasts comprising the fibrotic reticulum in idiopathic pulmonary fibrosis. The American journal of pathology 184, 1369–1383, doi: 10.1016/j.ajpath.2014.01.012 (2014). 24631025 PMC4005984

[b19] Yu C. C., Tsai C. H., Hsu H. I. & Chang Y. C. Elevation of S100A4 expression in buccal mucosal fibroblasts by arecoline: involvement in the pathogenesis of oral submucous fibrosis. PloS one 8, e55122, doi: 10.1371/journal.pone.0055122 (2013). 23383075 PMC3561403

[b20] Chang Y. C. . Activation of microRNA-494-targeting Bmi1 and ADAM10 by silibinin ablates cancer stemness and predicts favourable prognostic value in head and neck squamous cell carcinomas. Oncotarget 6, 24002–24016 (2015). 26090866 10.18632/oncotarget.4365 PMC4695166

[b21] Yu C. C., Chen P. N., Peng C. Y., Yu C. H. & Chou M. Y. Suppression of miR-204 enables oral squamous cell carcinomas to promote cancer stemness, EMT traits, and lymph node metastasis. Oncotarget 7, 20180–20192, doi: 10.18632/oncotarget.7745 (2016). 26933999 PMC4991446

[b22] Du R. . Hypoxia-induced Bmi1 promotes renal tubular epithelial cell-mesenchymal transition and renal fibrosis via PI3K/Akt signal. Molecular biology of the cell 25, 2650–2659, doi: 10.1091/mbc.E14-01-0044 (2014). 25009285 PMC4148254

[b23] Triviai I. . CD133 marks a stem cell population that drives human primary myelofibrosis. Haematologica 100, 768–779, doi: 10.3324/haematol.2014.118463 (2015). 25724578 PMC4450622

[b24] Liu S., Herault Y., Pavlovic G. & Leask A. Skin progenitor cells contribute to bleomycin-induced skin fibrosis. Arthritis Rheumatol 66, 707–713, doi: 10.1002/art.38276 (2014). 24574231

[b25] Zhang Q. . Tumor-like stem cells derived from human keloid are governed by the inflammatory niche driven by IL-17/IL-6 axis. PloS one 4, e7798, doi: 10.1371/journal.pone.0007798 (2009). 19907660 PMC2771422

[b26] Pant I., Kumar N., Khan I., Rao S. G. & Kondaiah P. Role of Areca Nut Induced TGF-beta and Epithelial-Mesenchymal Interaction in the Pathogenesis of Oral Submucous Fibrosis. PloS one 10, e0129252, doi: 10.1371/journal.pone.0129252 (2015). 26107172 PMC4479469

[b27] Das R. K. . Epithelio-mesenchymal transitional attributes in oral sub-mucous fibrosis. Experimental and molecular pathology 95, 259–269, doi: 10.1016/j.yexmp.2013.08.006 (2013). 23994666

[b28] Yang S. F., H. Y., Tsai C. H., Chou M. Y. & Chang Y. C. The upregulation of type I plasminogen activator inhibitor in oral submucous fibrosis. Oral oncology 39, 367–372 (2003). 12676256 10.1016/s1368-8375(02)00123-9

[b29] Tsai C. H., Yang S. F., Chen Y. J., Chou M. Y. & Chang Y. C. The upregulation of insulin-like growth factor-1 in oral submucous fibrosis. Oral oncology 41, 940–946, doi: 10.1016/j.oraloncology.2005.05.006 (2005). 16054426

[b30] Ni W. F., T. C., Yang S. F. & Chang Y. C. Elevated expression of NF-kB in oral submucous fibrosis-evidence for NF-kB induction by safrole in human buccal mucosal fibroblasts. Oral oncology 43, 557–562 (2007). 16996785 10.1016/j.oraloncology.2006.06.007

[b31] Chang Y. C., T. C., Tai K. W., Yang S. H., Chou M. Y. & Lii C. K. Elevated vimentin expression in buccal mucosal fibroblasts by arecoline *in vitro* as a possible pathogenesis for oral submucous fibrosis. Oral oncology 38, 425–430 (2002). 12110335 10.1016/s1368-8375(01)00083-5

[b32] Wight T. N. & Potter-Perigo S. The extracellular matrix: an active or passive player in fibrosis? American journal of physiology. Gastrointestinal and liver physiology 301, G950–G955, doi: 10.1152/ajpgi.00132.2011 (2011). 21512158 PMC3233785

[b33] Yang W. H., Deng Y. T., Hsieh Y. P., Wu K. J. & Kuo M. Y. Thrombin Activates Latent TGFbeta1 via Integrin alphavbeta1 in Gingival Fibroblasts. Journal of dental research 95, 939–945, doi: 10.1177/0022034516634288 (2016). 26912222

[b34] Yang W. H., Deng Y. T., Hsieh Y. P., Wu K. J. & Kuo M. Y. NADPH Oxidase 4 Mediates TGFbeta1-induced CCN2 in Gingival Fibroblasts. Journal of dental research 94, 976–982, doi: 10.1177/0022034515580986 (2015). 25858818

[b35] Liu Q. . Salvianolic Acid B Attenuates Experimental Pulmonary Fibrosis through Inhibition of the TGF-beta Signaling Pathway. Scientific Reports 6, 27610, doi: 10.1038/srep27610 (2016). 27278104 PMC4899783

[b36] Li H. Y. . Activation of TGF-beta1-CD147 positive feedback loop in hepatic stellate cells promotes liver fibrosis. Scientific Reports 5, 16552, doi: 10.1038/srep16552 (2015). 26559755 PMC4642271

[b37] Khan I., Kumar N., Pant I., Narra S. & Kondaiah P. Activation of TGF-beta pathway by areca nut constituents: a possible cause of oral submucous fibrosis. PloS one 7, e51806, doi: 10.1371/journal.pone.0051806 (2012). 23284772 PMC3526649

[b38] Chiou S. H. . Positive correlations of Oct-4 and Nanog in oral cancer stem-like cells and high-grade oral squamous cell carcinoma. Clinical cancer research: an official journal of the American Association for Cancer Research 14, 4085–4095, doi: 10.1158/1078-0432.CCR-07-4404 (2008). 18593985

[b39] Yu C. C. . miR145 targets the SOX9/ADAM17 axis to inhibit tumor-initiating cells and IL-6-mediated paracrine effects in head and neck cancer. Cancer research 73, 3425–3440, doi: 10.1158/0008-5472.CAN-12-3840 (2013). 23548270

