# Supplementary material for: Aberrant SSEA-4 upregulation mediates myofibroblast activity to promote pre-cancerous oral submucous fibrosis
Source: Sci Rep. 2016 Nov 15;6:37004. doi: 10.1038/srep37004 (PMC5109465; doi:10.1038/srep37004)
Supplement: Supplementary Information [file srep37004-s1.pdf]

Supplementary information

Aberrant SSEA-4 upregulation mediates myofibroblast activity to promote pre-cancerous oral submucous fibrosis

Cheng-Chia Yu[1,2,3], Chuan-Hang Yu[2,3], Yu-Chao Chang[2,3]*

[1]Institute of Oral Sciences, Chung Shan Medical University, Taichung, Taiwan;

[2]School of Dentistry, Chung Shan Medical University, Taichung, Taiwan;

[3]Department of Dentistry, Chung Shan Medical University Hospital, Taichung, Taiwan;

*Correspondence and requests for materials should be addressed to: Prof. Yu-Chao Chang, School of Dentistry, Chung Shan Medical University, email: cyc@csmu.du.tw

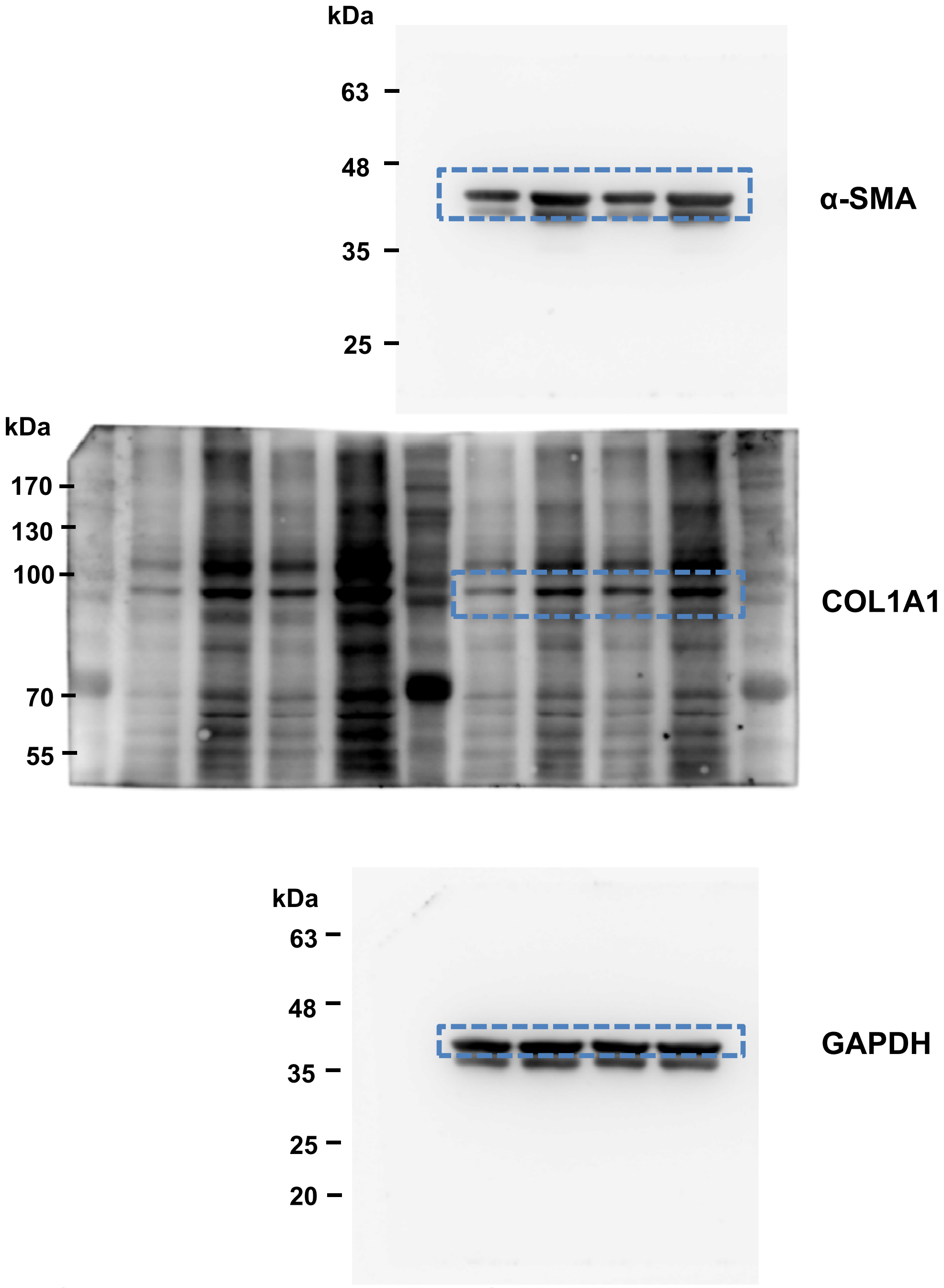

Supplementary Figure 1. Original immunoblotting data for Fig. 2

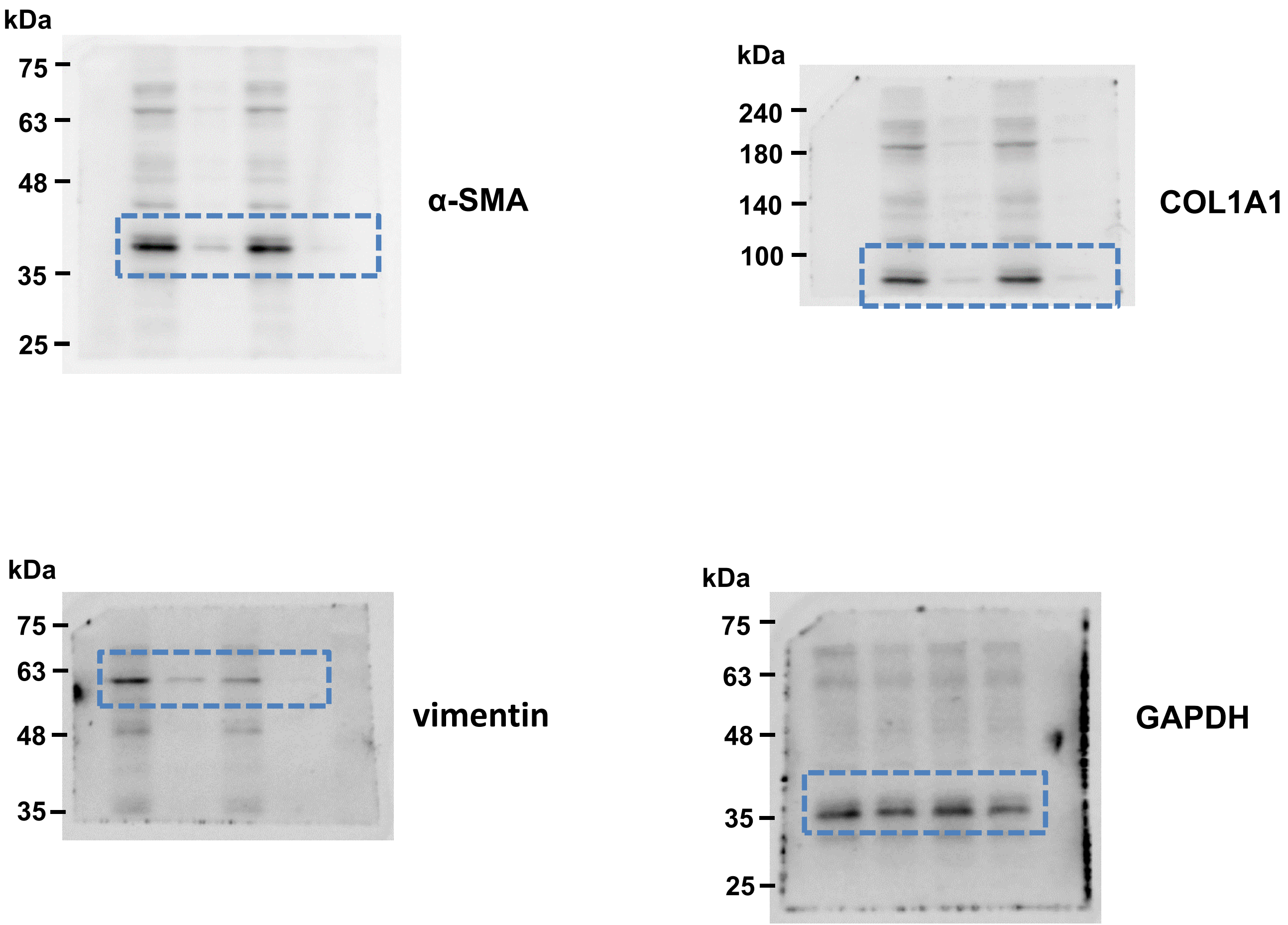

Supplementary Figure 2. Original immunoblotting data for Fig. 4
